# Mobile Phone Text Message Intervention on Diabetes Self-Care Activities, Cardiovascular Disease Risk Awareness, and Food Choices among Type 2 Diabetes Patients

**DOI:** 10.3390/nu11061314

**Published:** 2019-06-11

**Authors:** Martha J. Nepper, Jennifer R. McAtee, Lorey Wheeler, Weiwen Chai

**Affiliations:** 1 Nebraska Methodist Health System, 8111 Dodge Street, Omaha, NE 68114, USA; martha.nepper@nmhs.org; 2 Department of Nutrition and Health Sciences, University of Nebraska-Lincoln, 1700 N 35th Street, Lincoln, NE 68583, USA; jennifer.mcatee@huskers.unl.edu; 3 Nebraska Academy for Methodology, Analytics & Psychometrics, Nebraska Center for Research on Children, Youth, Families, and Schools, University of Nebraska-Lincoln, Lincoln, NE 68583, USA; lorey@unl.edu

**Keywords:** text messages, type 2 diabetes, diabetes self-care activities, cardiovascular disease risk awareness, food availability, food choices

## Abstract

This study examines the effects of educational text messages on diabetes self-care activities, cardiovascular disease (CVD) risk awareness, and home food availabilities related to food choices among patients with type 2 diabetes. Quasi-experimental design was used with 40 patients (58.0 ± 10.6 years) in the intervention group and 39 (55.7 ± 12.2 years) in the control group. In addition to the usual care provided for all participants, the intervention group received three educational text messages weekly for 12 weeks. Pre- and post-intervention measures were collected for both groups. Ninety-four percent of the participants receiving text messages indicated the usefulness of this program. The intervention group either maintained the same level or demonstrated small improvements in diabetes self-care activities after the intervention. Significant increases in scores of CVD risk awareness (57% increase; *p* = 0.04) and availabilities of fresh fruits (320% increase; *p* = 0.01) and fresh vegetables (250% increase; *p* = 0.02) in the home and weekly total (16% increase; *p* = 0.02) and moderate/vigorous (80% increase; *p* = 0.006) physical activity levels were observed for the intervention group relative to the control group. The pilot results suggest the feasibility and usefulness of the text message program for diabetes education. The study is registered with Clinical Trials.gov (NCT03039569).

## 1. Introduction

Type 2 diabetes is a complex and chronic illness affecting approximately 30.3 million people in the United States [1]. Adults with type 2 diabetes have a two to four-fold increase in the risk of developing cardiovascular disease (CVD), a leading cause of morbidity and mortality in this population [2]. Despite the strong link between type 2 diabetes and CVD, studies have found that patients with type 2 diabetes are unaware of their risk for developing CVD [3,4]. Nutrition and physical activity remain critical in the management of type 2 diabetes and are considered key in achieving optimal glycemic control and reducing major consequences such as CVD, foot damage, and kidney failure [5]. Since poor dietary practice may lead to insulin resistance, which further elevates blood glucose and lipid levels [6], self-inventory of household foods may offer a practical method for diabetes patients to monitor their food choices and dietary intake [7], thereby helping patients successfully manage the disease [6]. In addition to nutrition and physical activity, diabetes self-care activities include healthy coping skills, medication adherence, testing and managing blood glucose, problem solving, and strategies of reducing risk for health complications such as CVD [8]. These self-care activities have been used as a framework for patient-centered diabetes education.

Successfully managing type 2 diabetes requires life-long behavioral changes that can be challenging for many diabetes patients. Increasing evidence suggests that patients who take a more active role in their care achieve better health outcomes [9]. Conventional diabetes education, such as clinic visits with a health care provider, is commonly used for patients with type 2 diabetes to manage blood glucose and improve self-care skills. However, patients may have infrequent contact with health care providers because of their lack of time and transportation, expensive office visits and/or extended time between appointments. The use of text messages via cellular phones to convey health information and education represents a novel opportunity and low-cost method for improving diabetes self-care skills and increasing contact with health care providers. Previous work has demonstrated that text message interventions improve eating patterns, physical activity, and blood glucose management among patients with type 2 diabetes [10,11,12,13]. In addition, it has been shown that patients who receive short text messages find this type of intervention feasible and useful for managing the disease [12,13]. Evidence further suggests that cellular phone-based or web-based tools are particularly useful for patients living in rural regions with few specialized hospitals and limited access to health care clinics [13,14,15]. However, when addressing the effectiveness of using text messages for patients with type 2 diabetes, there is little research on whether a text message intervention would also increase the awareness of consequences of type 2 diabetes, for example, the risk of developing CVD. In addition, the impact of using text messages on patients’ home food environment such as the availability of healthy or unhealthy foods in the home has not yet been examined. The home food environment is particularly important to investigate for diabetes patients since it is likely to reflect patients’ food choices and purchase habits. In the current study, diabetes self-care education was performed using unidirectional text messages. Thus, the objective of the study was to examine the effect of educational text messages on diabetes self-care activities (general diet for healthy eating, specific diet for healthy eating, exercise, medication adherence, blood glucose testing, and foot care) and awareness of CVD risk among patients with type 2 diabetes. Additionally, the study also sought to assess whether the utilization of educational text messages had an influence on the availability of participants’ food choices in the home that were relevant to type 2 diabetes.

## 2. Materials and Methods

### 2.1. Participants

**Figure 1:**
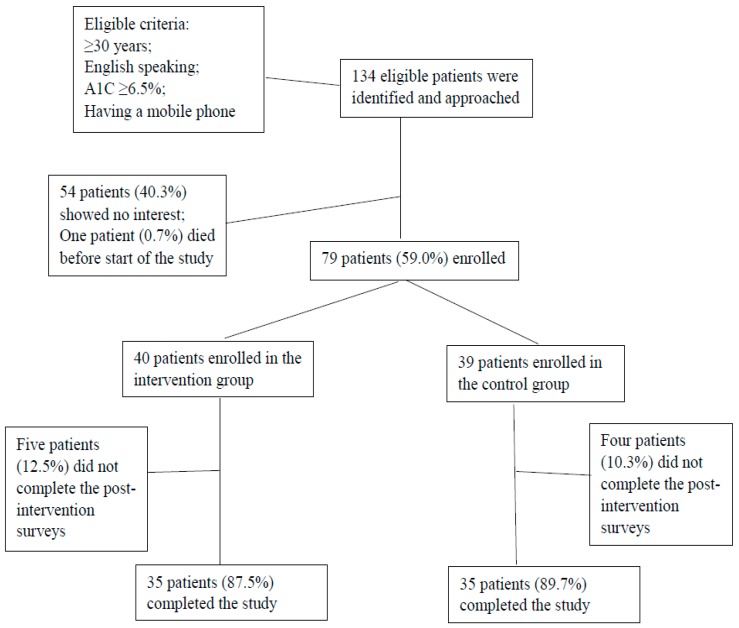
Flowchart of study participants.

Study participants were recruited from the Methodist Health System Center for Diabetes and Nutritional Health in Omaha, Nebraska, from February to December 2017. The center is an ambulatory outpatient clinic for treatment of patients with diabetes. Inclusion criteria were English-speaking adults with type 2 diabetes aged 30 years or older, self-reported hemoglobin A1C (HbA1C) greater than 6.5%, and having a cellular phone with the ability to receive text messages. Eligible participants who came into the clinic for outpatient diabetes care were identified and asked to participate in the study. All participants who elected to participate in the study were required to provide written informed consent. Seventy-nine patients (40 in the intervention group and 39 in the control group) with type 2 diabetes were enrolled in the study. Thirty-five participants in the intervention group and 35 participants in the control group completed the post-intervention surveys. Participants who did not complete the post-intervention surveys (*N* = 9) were either lost to follow-up, were no longer interested in participating, or did not respond to follow-up contact attempts (Figure 1). There were no differences in demographics and other factors relevant to type 2 diabetes between participants who completed the post-intervention surveys (*N* = 70) and those who did not (*N* = 9).

### 2.2. Study Design

A quasi-experimental design was used in this initial pilot study due to timeframe restraints. The intervention group (*N* = 40 patients) started approximately two months earlier than the control group (*N* = 39). The first 40 participants recruited were assigned to the intervention group. A survey regarding participants’ demographics and relevant risk factors for type 2 diabetes was completed by all the participants at baseline. Participants in both groups received the usual care for type 2 diabetes including an initial visit and follow-up visits from either a registered dietitian or a certified diabetes educator. The intervention group received three different educational text messages weekly (on Monday, Wednesday, and Friday) for 12 weeks (36 text messages in total). The text messages were sent in the late morning or early afternoon (between 11:00 am and 2:00 pm). The message topics were different each week during the first six weeks (Weeks 1 to 6; 18 text messages total) and repeated for the remaining weeks (Weeks 7 to 12; 18 text messages total). The messages consisted of strategies for healthy eating, being physically active, improving diabetes self-care skills including testing and managing blood glucose, taking medication, and increasing awareness of the risk of diabetes complications such as CVD. The text messages developed by the primary investigators were derived from the wording, topics, and guidelines provided by the American Association of Diabetes Educators (AADE7^TM^) [16]. Each text message was comprised of a short message and a link (which was a novel approach) to a specific AADE7^TM^ handout that allowed participants to open and retrieve the specific AADE7^TM^ information (Table 1). Text message contents were not piloted before the start of the study. However, the AADE7^TM^ handouts are available to the patients of the Methodist Health System Center for Diabetes and Nutritional Health as part of their usual care and diabetes education. Unidirectional text messages were sent by the project investigators to the participants in the intervention group via a computer-based text message program through a password protected computer which was only accessed by the investigators. Participants’ phone numbers used in the intervention were kept confidential and participants were advised not to reply to the text messages. If participants had a medical concern, they were advised to contact their physician or call 911. The control group (usual care group) did not receive text messages. Participants in both the intervention group (usual care and receiving text messages) and the control group (usual care only) completed surveys regarding their diabetes self-care activities, dietary intake, physical activity, awareness of CVD risk, and self-inventory of household foods at baseline and at the 12-week follow-up (conclusion of the intervention). The participants in the intervention group completed an additional survey to evaluate their satisfaction with receiving educational text messages for managing diabetes after the intervention was concluded. A $25 gift card was offered to all study participants. The research project was approved by the Institutional Review Boards of the University of Nebraska-Lincoln and Nebraska Methodist Health System. The study is registered with Clinical Trials.gov (NCT03039569).

### 2.3. Outcome Measures

#### 2.3.1. Diabetes Self-Care Activities

Diabetes self-care activities were measured using a previously validated Summary of Diabetes Self-Care Activities including categories of general diet for healthy eating (following a healthful eating plan; following one’s eating plan), specific diet for healthy eating (eating ≥5 servings of fruits and vegetables; avoiding consuming high-fat foods), exercise (participating in at least 30 minutes of physical activity per day; participating in a specific exercise session), testing blood glucose (testing blood glucose; testing blood glucose the number of times recommended by one’s health care provider per day), medication adherence (taking one’s recommended diabetes medication); and foot care (checking one’s feet; inspecting the inside of one’s shoes; washing one’s feet; avoiding soaking one’s feet; drying between one’s toes after washing). Respondents reported on the frequency with which they performed various self-care activities over the past seven days (how many days per week) [17]. The responses were scored from 0–7 accordingly; reverse scoring was used for a negative item. Individual items in each of the diabetes self-care activity categories (general diet for healthy eating, specific diet for healthy eating, exercise, blood glucose testing, medication adherence, and foot care) were combined to create an average score for the respective category.

#### 2.3.2. Dietary Intake and Physical Activity

In addition to diet and exercise items included in the aforementioned diabetes self-care activities, we additionally measured individual’s dietary intake and physical activity. Participants’ dietary intake was measured using a previously validated Block Fat-Sugar-Fruit-Vegetable Screener [18]. This screener contained 55 questions about frequency of food eaten (none or less than one day, one day, two days, three to four days, five to six days, or every day/per week) and portion sizes of 32 food items during the past month. Daily nutrient intakes including total calories were determined based on the data from the screener. Weekly physical activity levels were measured using the Block Physical Activity Screener [19]. This brief screening tool contained 11 items including job-related as well as daily life and leisure activities based upon National Human Activities Patterns Survey data. Total metabolic equivalent of task (MET) minutes per week for all the activities as well as for moderate/vigorous physical activities were calculated using the Ainsworth Compendium [20].

#### 2.3.3. CVD Risk Awareness

The CVD risk awareness questions were derived from a questionnaire used in a previous study [21]. Three questions were asked about how seriously a participant was concerned about having a CVD event in the next five years and in their lifetime (level of concern of CVD risk). The responses to the questions were scored from 0–3, indicating “no concern”, “low-level of concern”, “somewhat concerned”, and “highly concerned”, respectively. In this study, we summed the response scores for these three questions and calculated the mean score for the category. There was an additional question about how often a participant had a concern about having a CVD event with responses including “never” (zero times per week), “rarely” (one to two times per week), “sometimes” (three to four times per week), and “always” (five to seven times per week).

#### 2.3.4. Home Food Self-Inventory

A previously validated home food self-inventory checklist was used to assess the presence and absence of foods relevant to obesity and type 2 diabetes [22]. The checklist contained a total of 65 healthy and unhealthy food and beverage items including sweet and savory snacks, beverages, breakfast cereal/oatmeal, breads/pastas, dairy foods, and individual fruits and vegetables. Of these 65 food and beverage items/categories, there were 19 fruit and 16 vegetable items. Each fruit or vegetable item includes its fresh, canned/jarred/dried, and frozen forms. A “yes/no” format was used to indicate the availability of the food in the home with “1” indicating “yes” and “0” indicating “no”. The classification of “healthy” and “unhealthy” foods and beverages were derived from previous home food inventory tools [23,24] and followed the “We Can: Go, Slow, Whoa” food system, in which “Go” foods were considered healthy and “Whoa” foods were unhealthy [25].

Food items on the checklist were grouped into the following categories: all fruits, fresh fruits, canned/jarred/dried fruits, frozen fruits, all vegetables, fresh vegetables, canned/jarred/dried vegetables, frozen vegetables, all healthy foods (including fruits and vegetables), and all unhealthy foods. In addition, we also categorized foods on the checklist into high, medium, and low glycemic index (GI) foods according to the American Diabetes Association guidelines: low GI foods, GI ≤55; medium GI foods, GI ranging from 56 to 69; high GI foods, GI ≥70 [26]. GI measures how a food that contains carbohydrate influences blood glucose in comparison to a reference food (e.g., glucose or white bread) [26]. Similarly, the availability scores for food items in each of the above food categories were summed and the average score was calculated for the category.

### 2.4. Data Analysis

To make our quasi-experiment more rigorous, we followed recommendations by Shadish and colleagues [27], such as testing for baseline differences between groups and including the pre-test measure of outcomes to address selection bias resulting from not randomizing participants into groups. Preliminary analyses included comparing baseline characteristics between groups using *t*-tests for continuous variables and chi-square analyses for categorical variables. Our primary analyses included multivariate analysis of covariance (MANCOVA) to assess intervention effects by examining the differences between the intervention and the control groups at the 12 week follow-up. Any baseline differences between groups were controlled for in the test of effects at the 12 week follow-up by including the baseline measure of the outcome in the analyses. A proc GLM (generalized linear model) procedure was used to estimate MANCOVA. To control for experiment-wise error, the outcome/dependent variables were clustered into the following groups: diabetes self-care activities, fruit and vegetable availabilities, all healthy and unhealthy food availabilities, and GI-based fruit and vegetable availabilities. With MANCOVA, individual variables in each group mentioned above were analyzed together as group-based outcome/dependent variables (multivariate analysis). For outcome/dependent variables that were not categorized into a group (CVD risk awareness, MET minutes for total or moderate/vigorous physical activities, and intakes of dietary nutrients), analysis of covariance (ANCOVA) estimated by the Proc Glimmix procedure was used to assess the effects of intervention on these variables. We also used absolute change (time and treatment interaction), a more stringent test, to estimate intervention effects. Absolute change was determined as follows: absolute change = [(intervention group follow-up) – (intervention group baseline)] – [(control group follow-up) – (control group baseline)]. Since this is a pilot study, we used the results from both tests (MANCOVA/ANCOVA and absolute change) as supporting preliminary evidence for the intervention effects. Further, to provide perspective on the magnitude of the intervention effects, relative change, defined as (absolute change/intervention group baseline) x100%, was calculated. The covariates included in the models were age (continuous), sex, race/ethnicity (white, black, Hispanic, Asian, or other), education (college graduates or non-college graduates), baseline self-report HbA1C values, and the length of time of having had type 2 diabetes (<1 year, 1–5 years, or ≥5 years). For daily nutrient intake (carbohydrate, sugar, added sugar, total fat, saturated fat, and protein), we repeated the analyses with additional adjustment for total calorie intake and the results did not change substantially. An a priori power estimate suggests that our sample size (*N* = 35 in each group) was adequate for finding large effects (*d* = 0.8) and had 70% power for detecting medium effects (*d* = 0.5), assuming ɑ = 0.05 (two-tailed) based on Cohen’s recommendations [28]. SAS software version 9.4 (SAS Institute, Cary, NC, USA) was used for all analyses. We conservatively used two-tailed tests and *p* < 0.05 was considered statistically significant.

## 3. Results

### 3.1. Characteristics of Study Participants and Usefulness of Educational Text Messages

Overall, the mean ages of patients in the intervention and the control groups were 58.0 ± 10.6 and 55.7 ± 12.2 years, respectively. The majority of the participants were female (65% for the intervention group; 67% for the control group). The intervention and control groups had similar characteristics at baseline except for racial/ethnic distribution; a larger proportion of Hispanic participants was observed in the intervention group than the control group (*p* = 0.01). In addition, the intervention group had higher percentages of college graduates (49% vs. 34%) and those who had had type 2 diabetes for at least five years (73% versus 58%) compared to the control group (Table 2).

Participants in the intervention group (*N* = 35) completed a satisfaction survey regarding the feasibility and usefulness of the educational text messages in helping them with diabetes self-care management. The majority of the participants (94%) reported the text message intervention program was useful and stated that they would highly recommend this program to others with type 2 diabetes.

### 3.2. Diabetes Self-Care Activities, Dietary Intake, Physical Activity, and Awareness of CVD Risk

Overall, there were no statistically significant differences in changes of scores on diabetes self-care activities after the 12-week text message intervention. However, the intervention group in general maintained the same level or showed small improvements at the 12-week follow-up compared to the control group. In addition, weekly MET minutes for both in total (5548 versus 2877; 16% increase; *p* = 0.02) and moderate/vigorous physical activity (3163 versus 405; 80% increase; *p* = 0.006) were significantly higher for the intervention group than the control group at the 12-week follow-up after taking into account the baseline values for these variables. There were no significant changes in the intakes of relevant nutrients after intervention (Table 3).

With respect to CVD risk awareness, there was a statistically significant improvement in the score regarding how seriously a participant was concerned about having a CVD event (the level of concern) in the intervention group compared to the control group (57% increase; *p* = 0.04). However, the average score was nevertheless at the lower end of the scale (1.26), being between “low-level of concern” and “somewhat concerned” for the intervention group (Table 3). Similarly, at the baseline, a majority of the study participants in both groups reported that they were never or rarely concerned about a CVD event (70% for the intervention group; 68% for the control group). After 12 weeks of intervention, “never” or “rarely concerned” about CVD risk was reported by 68% of the participants in the intervention group and 82% of those in the control group.

### 3.3. Home Food Availabilities Related to Food Choices

The intervention group had significant increases in availability scores for fresh fruits (320% increase; *p* = 0.01) and fresh vegetables (250% increase; *p* = 0.02) in the home after the intervention compared to the control group. When the food availabilities were assessed based on GI values, there was a significant increase in the score for high GI fruit availability (431% increase; *p* = 0.001) and a decrease in the score for medium GI vegetable availability (40% decrease; *p* = 0.03) for the intervention group relative to the control group at the 12-week follow-up. It appeared that high GI vegetables were more likely to be available in the home among participants in both groups (Table 4).

## 4. Discussion

Using text messages via a cellular phone device is a low-cost and simple method of delivering health information and education. In this pilot study, the intervention group either maintained the same level or showed small improvements in diabetes self-care activities after 12 weeks of the text message intervention. Improvements in adherence to following a specific diet plan for diabetes [29], eating habits [30], physical activity [13,30], and self-care management skills [29] among type 2 diabetes patients using text messages have been documented previously. In the current study, each patient in the intervention group received a short text message three days (one message per day) per week. Each text message also had a link that directed patients to the AADE7^TM^ handout to provide patients additional information and strategies of diabetes self-care skills, which was a novel approach to diabetes education. Based on the feedback from study participants, the current educational text message program was perceived as useful and beneficial (94% responded that yes it was) for helping type 2 diabetes patients with self-care management, suggesting the feasibility and usefulness of the program.

There are possible explanations for the non-statistically significant improvements in diabetes self-care activities. First, patients in both intervention and control groups had been receiving the usual care for type 2 diabetes (clinic visits with registered dietitians or certified diabetes educators) and therefore might have already been working on their self-care management skills before the intervention. This was suggested by the baseline data showing patients in both groups having an average of five days or more per week of engaging in self-care activities such as following eating plans, checking blood glucose levels, and taking medications. Thus, the positive changes due to the text message intervention would be more substantial for diabetes patients who do not receive routine care for the disease, such as those living in rural areas with limited access to health care or having other conditions resulting in infrequent contacts with health care providers or diabetes educators. Second, the participants in the control group were not prohibited from seeking diabetes self-care and other health information online or through other resources, thereby potentially mitigating differences from the text message intervention. Third, the relatively short intervention period (12 weeks) may have also contributed to non-statistically significant results. Promisingly, our results (from the ANCOVA tests) suggest significant improvements in weekly MET minutes for both total and moderate/vigorous physical activity in the intervention group compared to the control group at the 12-week follow-up. Although the results from the more stringent test for absolute change were not statistically significant, given the pilot study nature, the significant findings based on ANCOVA nevertheless provide preliminary evidence for the positive effect of the current intervention program on patients’ physical activity levels as each participant in the program received six messages total on physical activity.

It is established that adults with type 2 diabetes have a two- to four-fold increase in the risk of developing CVD; however, the majority of study participants in the intervention and the control groups did not have a high awareness of CVD risk at baseline. This finding was consistent with previous studies [3,4] that reported adults with type 2 diabetes were unaware of their risk for developing CVD. The intervention group indeed had a significant improvement in the score regarding the level of a participant’s concern over having a CVD event after the intervention. However, despite the increase, the average score was still at the lower end of the scale between “low-level of concern” and “somewhat concerned” for the intervention group after receiving text messages for 12 weeks. In addition, being “never” or “rarely concerned” about CVD risk was reported by most of the participants after the intervention (68% for the intervention group and 82% for the control group). In the current study, the intervention group received six messages total on the topic of reducing the risk of complications associated with diabetes. Although each message included a link to an AADE7^TM^ handout addressing the direct relationship between type 2 diabetes and CVD risk, it is possible that participants did not click on the added link in the text messages to learn more about this information. Therefore, future interventions using educational text messages should focus more on increasing participants’ awareness of CVD risk. For example, when creating text messages, one may consider phrasing the messages with extra emphasis on the strong link between type 2 diabetes and CVD. Furthermore, extending the intervention period from 12 weeks to six months and including more text messages on the topic may enhance the impact of intervention on CVD risk awareness. Nevertheless, the significant improvement, although small as observed in this study, suggests that the current text message program to some extent made participants aware of CVD risk, a first step towards achieving the ultimate goal that is to reduce the risk of developing CVD and other diabetes-related complications.

The results from the current study suggest that the 12-week text message intervention had promising effects on the participants’ food choices that were reflected by the presence or absence of foods relevant to type 2 diabetes in the home. The study observed significant increases in the availability scores for fresh fruits and fresh vegetables in the intervention group after receiving educational text messages for 12 weeks. Although the availability of high GI fruits also increased after the intervention and the participants in both groups were more likely to store high GI vegetables in the home, we should not make dietary recommendations for healthy eating solely based on GI values since GI itself does not reflect the likely quantity an individual would eat and high GI fruits and vegetables contain other beneficial compounds such as fiber, vitamins, minerals, and polyphenols. Future diabetes educational programs using text messages should educate patients on the health benefits of increasing fruit and vegetable intake (e.g., fiber, vitamins, minerals, and polyphenol content). In addition, educational messages should address the influence of fruit and vegetable intake on blood glucose levels when eaten in the appropriate portion sizes to help patients make wise food choices, since GI does not address portion sizes which are relevant for managing blood glucose levels.

Our study had limitations. The non-randomized, quasi-experimental study design may have increased baseline differences between the intervention and the control groups due to selection bias. However, there were no major differences in the relevant characteristics at baseline between the two groups and the current analyses were adjusted for the relevant covariates to address potential selection bias. Participants who agreed to enroll in the study may have been more interested in improving their diabetes self-care skills and healthy habits relative to those who did not. There is a possibility that participants in the intervention group deleted the text messages without reading the message or clicking on the AADE7^TM^ handout link or did not receive some text messages, thus negating any significant health behavior changes. However, all possible attempts were made to ensure that the participants were receiving and reading the messages. For example, investigators called participants several times during the study and visited with them when they came into the clinic on whether they were receiving the text messages or had any problems with opening the AADE7^TM^ handout link. During the study, no problems of undeliverable messages were encountered. The results might be underestimated or overestimated due to loss at follow-up that occurred in the study. However, there were no differences in demographics and relevant factors at baseline between participants who completed the post-intervention surveys and those who did not. In addition, participants might know when to “expect” the messages, which may have some effects on the effectiveness of the intervention. Lastly, although validated self-report measures were used, objective indicators may be more accurate for assessing the intervention effects.

## 5. Conclusions

The results from this pilot study suggest the feasibility and usefulness of using educational text messages for patients with type 2 diabetes to maintain or improve their diabetes self-care skills. Further, the current text message program can benefit patients living in rural areas with limited access to health care or having other conditions resulting in infrequent contacts with health care providers.

The pilot results also demonstrate a small but statistically significant increase in CVD risk awareness as well as significant increases in physical activity and the availabilities of fresh fruits and vegetables in the home among participants receiving text messages. Although these results need to be confirmed by randomized experimental trials in the future, our findings, especially the ones related to CVD risk awareness and home food self-inventory, add to the growing body of literature on using text messages to deliver health information to patients with health concerns, including type 2 diabetes. For future interventions, approaches such as extending the length of the intervention, increasing the frequency of delivering such messages to participants or combining with other strategies such as a telephone-based coaching approach may enhance the impact of the program. When revising the content of the educational messages, one may need to increase the focus on reducing CVD risk by highlighting the direct relationship between type 2 diabetes and CVD. Also, text messages should address the importance of including fruits and vegetables in a patient’s daily food intake for health benefits, and how portion sizes influence blood glucose levels, which may help patients make healthy food choices.

## Figures and Tables

**Table 1:** Contents of educational text messages for type 2 diabetes patients.

AADE7^TM^ Handout Titles/Topics	Contents of Text Messages ^a^
Healthy Eating (Weeks 1 and 7)	1) Eat breakfast every day!
	2) There are only three main types of nutrients in foods: carbohydrates, proteins, and fats. A healthy meal will include all three of these.
	3) Do not skip meals! Remember to eat regular meals and snacks every day.
	*For more info: https://www.diabeteseducator.org/patient-resources/aade7-self-care-behaviors/healthy-eating*
Healthy Coping (Weeks 2 and 8)	1) Think positive! Feeling down? Remember your successes and feel good about your progress with diabetes.
	2) Build healthy relationships. You are not alone when you have diabetes.
	3) If you are sad, anxious or stressed, go for a walk or stand up and stretch.
	*For more info: https://www.diabeteseducator.org/patient-resources/aade7-self-care-behaviors/healthy-coping*
Monitoring (Weeks 3 and 9)	1) Checking your blood sugars gives you vital information about your diabetes control.
	2) Monitoring your blood sugars helps you know when they are on target.
	3) Call your doctor or diabetes educator if you are concerned about your blood sugars.
	*For more info: https://www.diabeteseducator.org/patient-resources/aade7-self-care-behaviors/ aade7-self-care-behaviors-monitoring*
Being Active (Weeks 4 and 10)	1) Being active has many health benefits, like improving blood pressure and blood sugars.
	2) If you haven’t exercised for a while, start with a five minute walk and increase gradually.
	3) Break activity into three ten minute sessions.
	*For more info: https://www.diabeteseducator.org/patient-resources/aade7-self-care-behaviors/being-active*
Taking Medication and Problem Solving (Weeks 5 and 11)	1) Take notes when you visit with your doctor about your medication.
	2) Learn what causes your blood sugar to go above or below target.
	3) Talk to your doctor about how to improve your blood sugar.
	*For more info: https://www.diabeteseducator.org/patient-resources/aade7-self-care-behaviors/ taking-medication* *https://www.diabeteseducator.org/patient-resources/aade7-self-care-behaviors/problem-solving*
Reducing Risks (Weeks 6 and 12)	1) See your eye doctor at least once a year
	2) Keep a wallet card that lists all of the tests you should be regularly getting and the targets for each.
	3) Lowering your cholesterol can decrease your risk for a stroke. Talk to your doctor about what you can do.
	*For more info: https://www.diabeteseducator.org/patient-resources/aade7-self-care-behaviors/reducing-risks*

^a^ Each text message included a link to a specific American Association of Diabetes Educator (AADE7^TM^) handout (36 text messages total).

**Table 2:** Baseline characteristics of study participants with type 2 diabetes ^a.^

Characteristics	Intervention	Control	*p* Value ^b^
*N*	40	39	
Age (year)	58.0 ± 10.6	55.7 ± 12.2	0.21
Sex (%)			0.82
Male	34.7	32.9	
Female	65.3	67.1	
Race/ethnicity (%)			0.01
White	84.0	93.2	
Black	2.7	5.4	
Hispanic	8.0	0	
Asian	2.7	0	
Other	2.7	1.4	
BMI (kg/m^2^)	34.6 ± 9.05	35.9 ± 6.1	0.30
Hemoglobin A1C (%) ^c^	7.8 ± 1.4	8.2 ± 1.9	0.09
College graduate (%)	49.3	33.8	0.05
Having diabetes <1 year (%)	12.0	20.3	0.17
Having diabetes ≥5 years (%)	73.3	58.1	0.05
Taking diabetes medication (%)	92.0	91.9	0.98
Current smoker (%)	4.0	8.0	0.30

^a^ Data are given as mean ± standard deviation unless otherwise specified. ^b^ *p* value for difference between the intervention and control groups by *t* test for continuous variables and chi-square test for categorical variables. ^c^ Hemoglobin A1C values were based on self-report values by study participants.

**Table 3:** Diabetes self-care activities, cardiovascular disease (CVD) risk awareness, physical activity (PA) and dietary intake at baseline and 12-week follow-up.

Variable	Baseline		12-Week Follow-Up		Absolute Change ^a^		Relative Change ^b^
	Mean (SE)^c^	*p* ^d^	Mean (SE) ^c^	*p* ^d^	Mean (SE)^c^	*p* ^e^	
**Diabetes Self-Care Activities (day/week)**							
General diet for healthy eating							
Intervention	4.66 (0.43)	0.14	5.18 (0.46)	0.61	0.76 (0.52)	0.15	16%
Control	5.22 (0.46)		4.98 (0.48)				
Specific diet for healthy eating							
Intervention	4.20 (0.40)	0.93	4.03 (0.43)	0.62	−0.21 (0.48)	0.67	−5%
Control	4.17 (0.43)		4.21 (0.45)				
Exercise							
Intervention	5.14 (0.61)	0.67	4.45 (0.65)	0.77	−0.07 (0.74)	0.93	−1%
Control	4.91 (0.65)		4.29 (0.68)				
Blood glucose testing							
Intervention	5.06 (0.62)	0.08	5.28 (0.66)	0.49	0.57 (0.75)	0.45	11%
Control	6.01 (0.67)		5.66 (0.69)				
Medication adherence							
Intervention	6.05 (0.35)	0.51	6.35 (0.38)	0.09	0.33 (0.42)	0.44	5%
Control	5.85 (0.38)		5.82 (0.39)				
Foot care							
Intervention	4.23 (0.41)	0.79	4.86 (0.43)	0.08	0.53 (0.49)	0.28	13%
Control	4.13 (0.43)		4.23 (0.45)				
**CVD Risk Awareness**							
Intervention	1.01 (0.23)	0.21	1.26 (0.25)	0.13	0.58 (0.29)	0.04	57%
Control	1.27 (0.25)		0.94 (0.26)				
**PA (MET** ^f^ **minute/week)**							
Total PA							
Intervention	4806 (1451)	0.08	5548 (1645)	0.02	768 (1533)	0.62	16%
Control	2904 (1541)		2877 (1602)				
Moderate/vigorous PA							
Intervention	2112 (1435)	0.25	3163 (1555)	0.006	1688 (1300)	0.20	80%
Control	1042 (1430)		405 (1532)				
**Dietary Intake**							
Total calories (kcal/day)							
Intervention	1598 (152)	0.43	1499 (161)	0.79	−134 (176)	0.45	−8%
Control	1417 (161)		1452 (9168)				
Carbohydrates (g/day)							
Intervention	162.3 (17.3)	0.54	153.4 (18.2)	0.81	−12.5 (20.0)	0.53	−8%
Control	140.2 (18.3)		143.9 (19.1)				
Total sugar (g/day)							
Intervention	58.9 (8.4)	0.52	52.4 (8.9)	0.50	0.4 (9.7)	0.96	1%
Control	49.6 (8.9)		44.7 (9.3)				
Added sugar (g/day)							
Intervention	34.8 (7.3)	0.39	29.6 (7.7)	0.25	2.1 (8.4)	0.80	6%
Control	30.1 (7.7)		22.9 (8.0)				
Total fat (g/day)							
Intervention	67.3 (7.0)	0.28	61.1 (7.4)	0.89	−7.0 (8.1)	0.39	−10%
Control	60.1 (7.4)		60.9 (7.7)				
Saturated fat (g/day)							
Intervention	23.1 (2.5)	0.30	21.0 (2.6)	0.98	−2.1 (2.9)	0.47	−9%
Control	21.3 (2.7)		21.3 (2.8)				
Protein (g/day)							
Intervention	70.4 (6.9)	0.71	68.3 (7.2)	0.62	−5.1 (7.9)	0.53	−7%
Control	62.4 (7.3)		65.3 (7.6)				

^a^ Absolute change = [(intervention group follow-up) – (intervention group baseline)] – [(control group follow-up) – (control group baseline)]. ^b^ Relative change = (absolute change / intervention group baseline) x 100%. ^c^ Adjusted mean is presented. ^d^ *p* value for difference between the intervention and the control groups by MANCOVA or ANCOVA adjusting for age, sex, race/ethnicity, education, self-report hemoglobin A1C, and length of time having had type 2 diabetes at baseline. ^e^ *p* value for absolute change adjusting for age, sex, race/ethnicity, education, self-report hemoglobin A1C, and length of time having had type 2 diabetes at baseline. ^f^ MET = metabolic equivalent of task.

**Table 4:** Availability of fruits and vegetables in the home at baseline and 12-week follow-up.

Food Availability	Baseline		12-Week Follow-Up		Absolute Change ^a^		Relative Change ^b^
	Mean (SE) ^c^	*p* ^d^	Mean (SE) ^c^	*p* ^d^	Mean (SE) ^c^	*p* ^e^	
**Fruits**							
Total							
Intervention	0.06 (0.04)	0.29	0.08 (0.04)	0.001	0.04 (0.03)	0.15	67%
Control	0.03 (0.04)		0.01 (0.04)				
Fresh							
Intervention	0.05 (0.09)	0.94	0.17 (0.08)	0.0005	0.16 (0.06)	0.01	320%
Control	0.05 (0.09)		0.01 (0.09)				
Canned/jarred/dried							
Intervention	0.06 (0.06)	0.15	0.04 (0.05)	0.35	−0.02 (0.04)	0.63	−33%
Control	0.01 (0.06)		0.02 (0.06)				
Frozen							
Intervention	0.05 (0.04)	0.18	0.02 (0.04)	0.27	−0.008 (0.03)	0.78	−16%
Control	0.02 (0.04)		−0.005 (0.04)				
**Vegetables**							
Total							
Intervention	0.13 (0.05)	0.79	0.11 (0.05)	0.98	−0.009 (0.04)	0.82	−7%
Control	0.12 (0.05)		0.11 (0.05)				
Fresh							
Intervention	0.06 (0.09)	0.94	0.18 (0.09)	0.0008	0.15 (0.07)	0.02	250%
Control	0.06 (0.09)		0.02 (0.09)				
Canned/jarred/dried							
Intervention	0.06 (0.06)	0.12	0.05 (0.05)	0.31	−0.02 (0.04)	0.62	−33%
Control	0.01 (0.05)		0.02 (0.06)				
Frozen							
Intervention	0.04 (0.03)	0.21	0.02 (0.03)	0.11	0.003 (0.02)	0.90	8%
Control	0.02 (0.03)		−0.005 (0.03)				
**All healthy foods** ^f^							
Intervention	1.00 (0.06)	0.45	1.08 (0.06)	0.68	0.01 (0.06)	0.82	1%
Control	1.04 (0.06)		1.10 (0.06)				
**All unhealthy foods**							
Intervention	1.41 (0.09)	0.83	1.50 (0.09)	0.75	0.04 (0.10)	0.70	3%
Control	1.42 (0.09)		1.47 (0.10)				
**Based on Glycemic Index (GI)** ^g^							
**Fruits**							
Low GI							
Intervention	11.03 (2.15)	0.25	10.68 (2.17)	0.05	1.43 (2.35)	0.54	13%
Control	9.06 (2.23)		7.28 (2.34)				
Medium GI							
Intervention	14.75 (3.15)	0.43	17.51 (3.17)	0.004	5.27 (3.43)	0.13	36%
Control	12.78 (3.27)		10.27 (3.42)				
High GI							
Intervention	8.07 (9.68)	0.02	40.02 (9.75)	0.03	34.79 (10.54)	0.001	431%
Control	25.96 (10.04)		23.13 (10.50)				
**Vegetables**							
Low GI							
Intervention	8.90 (1.34)	0.77	9.09 (1.35)	0.69	0.74 (1.46)	0.61	8%
Control	9.31 (1.39)		8.67 (1.45)				
Medium GI							
Intervention	47.83 (7.85)	0.67	32.24 (7.91)	0.009	−19.33 (0.03)	0.03	−40%
Control	45.13 (8.15)		48.87 (8.52)				
High GI							
Intervention	91.58 (15.60)	0.90	65.08 (15.72)	0.08	−23.68(16.99)	0.17	−26%
Control	90.01 (16.20)		87.20 (16.93)				

^a^ Absolute change = [(intervention group follow-up) – (intervention group baseline)] – [(control group follow-up) – (control group baseline)]. ^b^ Relative change = (absolute change / intervention group baseline) x 100%. ^c^ Adjusted mean is presented. ^d^ *p* value for difference between the intervention group and the control group by MANCOVA adjusting for age, sex, race/ethnicity, education, self-report hemoglobin A1C, and length of time having had type 2 diabetes at baseline. ^e^ *p* value for absolute change adjusting for age, sex, race/ethnicity, education, self-report hemoglobin A1C, and length of time having had type 2 diabetes at baseline. ^f^ Including fruits and vegetables. ^g^ Low GI foods: GI ≤55; medium GI foods: GI between 56–69; high GI foods: GI ≥70.
